# Alterations of the Blood-Brain Barrier and Regional Perfusion in Tumor Development: MRI Insights from a Rat C6 Glioma Model

**DOI:** 10.1371/journal.pone.0168174

**Published:** 2016-12-22

**Authors:** Monika Huhndorf, Amir Moussavi, Nadine Kramann, Olga Will, Kirsten Hattermann, Christine Stadelmann, Olav Jansen, Susann Boretius

**Affiliations:** 1 Clinic of Radiology and Neuroradiology, University Medical Center, Schleswig Holstein, Christian Albrechts University, Kiel, Germany; 2 Molecular Imaging North Competence Center, Christian Albrechts University, Kiel, Germany; 3 German Primate Center, Leibniz Institute for Primate Research, Göttingen, Germany; 4 Institute of Neuropathology, University Medical Center Göttingen, Germany; 5 Institute of Anatomy, Christian Albrechts University, Kiel, Germany; 6 Faculty for Biology and Psychology, Goettingen University, Goettingen, Germany; George Washington University, UNITED STATES

## Abstract

**Objectives:**

Angiogenesis and anti-angiogenetic medications play an important role in progression and therapy of glioblastoma. In this context, *in vivo* characterization of the blood-brain-barrier and tumor vascularization may be important for individual prognosis and therapy optimization.

**Methods:**

We analyzed perfusion and capillary permeability of C6-gliomas in rats at different stages of tumor-growth by contrast enhanced MRI and dynamic susceptibility contrast (DSC) MRI at 7 Tesla. The analyses included maps of relative cerebral blood volume (CBV) and signal recovery derived from DSC data over a time period of up to 35 days after tumor cell injections.

**Results:**

In all rats tumor progression was accompanied by temporal and spatial changes in CBV and capillary permeability. A leakage of the blood-brain barrier (slow contrast enhancement) was observed as soon as the tumor became detectable on T2-weighted images. Interestingly, areas of strong capillary permeability (fast signal enhancement) were predominantly localized in the center of the tumor. In contrast, the tumor rim was dominated by an increased CBV and showed the highest vessel density compared to the tumor center and the contralateral hemisphere as confirmed by histology.

**Conclusion:**

Substantial regional differences in the tumor highlight the importance of parameter maps in contrast or in addition to region-of-interest analyses. The data vividly illustrate how MRI including contrast-enhanced and DSC-MRI may contribute to a better understanding of tumor development.

## Introduction

Glioblastoma multiforme (GM) is one of the most malignant and frequent primary brain tumors [1]. Current therapies combine surgery, chemotherapy and radiation, but the mean survival time of GM is only 14.6 months [2]. The characterization of tumor microvasculature including cerebral blood volume and vascular permeability is essential in glioblastoma diagnostics, in monitoring of the therapeutic response, and in therapeutic research particularly of anti-angiogenic medications [3, 4]. Dynamic susceptibility contrast-enhanced (DSC) MRI is commonly used to measure cerebral blood volume (CBV) and several DSC-MRI studies have shown a correlation of higher CBV values with higher glioma grades [5, 6].

Tumor-driven angiogenesis and inflammation are often accompanied by increased capillary permeability. This is detected by contrast agents such as Gd-DTPA passing into the extravascular space. Compared to the initial intravascular bolus, however, the concentration of the extravascular contrast agent is significantly lower and increases slowly over time leading to a signal enhancement on T1-weighted images. This effect is utilized by dynamic contrast-enhanced (DCE) MRI [7, 8].

**Fig 1:**
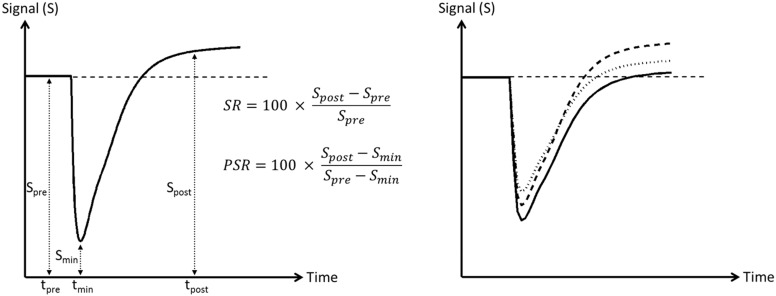
Simulated signal-intensity time curve after administration of the contrast agent. Left: schematic illustration of signal recovery (SR) and percentage of signal recovery (PSR). SR is defined as the difference between the signal intensity immediately after the first pass of the contrast agent (S_post_ at t_post_, in humans usually 60 s after bolus arrival) and the pre-contrast (S_pre_) signal intensity, while PSR is given by the difference of the signal intensity at t_post_ to the minimum of the signal intensity-curve (S_min_) divided by the difference between pre-contrast (S_pre_) and minimum (S_min_) signal intensity. Right: Influence of TR and TE on the signal-intensity time curve. The stronger the T1-weighting (reduction of TR) and the weaker the T2*- weighting (reduction of TE) the higher S_min_ and S_post_ for the identical time curve of the contrast agent concentration (solid line: TR/TE = 1500/50 ms, dashed line: TR/TE = 1200/50 ms, dotted line: TR/TE = 1500/40 ms assuming T1/T2 = 1000/100 ms and r1/r2 = 4/5 l mmol^-1^ s^-1^).

In clinical diagnostics of humans, capillary permeability imaging has often been limited to the analysis of a short time period after contrast agent injection or even only the first pass of the contrast agent. In particular, the percentage of signal recovery (PSR) has been widely used. PSR is the difference between the signal intensity at a defined time point (usually 60 s after bolus arrival in humans) and the minimum of the signal intensity curve (peak of the bolus) divided by the difference of the signal intensities at pre-contrast baseline and minimum (Fig 1). This method strongly depends on the applied MR-parameters repetition time (TR), echo time (TE), flip angle, and magnetic field strength (Fig 1), but PSR may carry valuable diagnostic information [9]. For instance, primary central nervous system (CNS) lymphomas often exceeded the baseline (PSR > 100%) whereas high-grade astrocytomas did not [10–12]. PSR may thus help differentiating between glioblastoma, metastases and primary CNS lymphoma [13, 14], showing highest PSR for lymphoma and lowest values for metastases. Moreover, PSR may differentiate between low and high grade gliomas [15].

C6-cell-glioma in rats mimics several features of human glioblastoma including high mitotic index, focal tumor necrosis, parenchymal invasion and neoangiogenesis [16–18]. So far, however, only few *in vivo* studies have evaluated the time course of perfusion properties in this model [19, 20].

Here, we optimized a DSC-MRI protocol for application at 7 T and small rodents. Maps of relative CBV and PSR were repeatedly obtained during tumor development in rats after intracranial inoculation of different numbers of C6 cells. In addition, a simple and robust map reflecting the signal intensity relative to baseline shortly after the first pass of the contrast agent is introduced and is referred to as signal recovery (SR) map in this presentation (Fig 1).

Being aware of the parameter dependency and limitations of PSR, the intention of this study was not to propose an entirely new approach to perfusion measurement but rather to characterize the perfusion properties of this brain tumor model using an MRI approach most similar to the methods commonly used in medical practice.

## Materials and Methods

### Rats

All studies were performed in accordance with German animal protection laws with the specific approval of the responsible governmental authority (Ministerium für Landwirtschaft, Umwelt u. ländliche Räume des Landes Schleswig-Holstein, V 312–72241.121–17 (36-3/12). Male Wistar rats (age 6–10 weeks, n = 5) were anesthetized by intraperitoneal injection of medetomidine (0.4 mg/kg) and ketamine (70 mg/kg). 5 μl of C6 cells with different cell counts (Table 1) were stereotactically injected into the basal ganglia (3 mm left of the bregma, 4.5 mm deep into the brain). The positioning of the injection was verified by T2-weighted MRI. To prevent postoperative pain 50 mg/kg metamizol was injected once subcutaneously. After tumor cell injections, rats underwent daily visual inspections using a comprehensive scoring system and stop criteria predefined and approved by the responsible governmental authority.

**Table 1:** Points in time at which a difference was observed (marked by + in a grey box) between the hemisphere of tumor cell injection and the contralateral hemisphere, shown separately for each MR parameter analyzed.

Number of tumor cells injected	Parameter	Day 5	Day 9	Day 14	Day 21	Day 27	Day 35
1,000	**T2w**				+	+	+
	**Gd-T1w**				+	+	+
	**SR**					+	+
	**PSR**					invalid	invalid
	**CBV**					invalid	invalid
10,000	**T2w**	+	+	+	+	dead	dead
	**Gd-T1w**	+	+	+	+		
	**SR**			+	+		
	**PSR**			+	+		
	**CBV**			+	+		
50,000	**T2w**		+	+	+	+	dead
	**Gd-T1w**		+	+	+	+	
	**SR**				+	+	
	**PSR**				+	+	
	**CBV**				+	+	
100,000	**T2w**	+	+	+	+	dead	dead
	**Gd-T1w**	+	+	+	+		
	**SR**			+	+		
	**PSR**			+	+		
	**CBV**			+	+		
500,000	**T2w**	+	+	+	+	dead	dead
	**Gd-T1w**	+	+	+	+		
	**SR**		+	+	+		
	**PSR**		+	+	+		
	**CBV**			+	+		

### MRI

On days 5, 9, 14, 21 (for lower cell counts also on days 27 and 35) after tumor cell injection the rats were anaesthetized by intraperitoneal injection of medetomidine and ketamine, subsequently intubated and maintained under anesthesia with isoflurane (0.5–1.5% in ambient air) using active ventilation. All MRI data sets were obtained at a field strength of 7 T (ClinScan^™^, Bruker BioSpin, Ettlingen, Germany) including anatomical T2-weighted images (2D FSE, three orthogonal directions, TR/TE = 3150/41 ms, 7 echoes, 125 x 125 x 500 μm^3^, field of view (FOV) = 40.0 x 32.5 mm^2^, 20 slices) and T1-weighted images (3D FLASH, TR/TE = 10/0.9 ms, flip angle 15, 170 x 170 x 170 μm^3^, FOV = 32.6 x 29.5 x 32.6 mm^3^). The latter were obtained before and 10 min after bolus injection of 0.2 mmol/kg Gd-DTPA (Magnograph^®^) via a tail vein catheter followed by 0.4 ml isotonic NaCl. DSC-MRI (2D EPI, TR/TE = 400/7.3 ms, flip angle 90°, 312.5 x 312.5 x 1000 μm^3^, FOV = 40 x 40 mm^2^, temporal resolution 400 ms/4 axially oriented slices, 400 repetitions) was continuously performed before, during and up to 150 s after injection of the contrast agent. The DSC-MRI parameter in combination with the concentration of the contrast agent were chosen in a way that, on average, the signal-intensity in healthy brain tissue (excluding larger veins) did not exceed the baseline and went back to baseline during the time of DSC-MRI data acquisition. For all measurements a 4-channel phased array coil was used for signal detection in combination with a quadrature birdcage coil for excitation (Bruker BioSpin, Ettlingen, Germany).

### Data analysis

Before analyzing the time course of DSC-MRI the data have been filtered by a Gauss filter (3 x 3, sigma = 0.5) along the spatial axes (in-plane) followed by a median filter (width: 5 data points) along the time axis. The concentration-time curve of the contrast agent was determined from the signal-time curve as described earlier [21]. To diminish effects of blood recirculation, a gamma-variate function was fitted to the concentration-time curve [22, 23] and the cerebral blood volume (CBV) was calculated pixel wise [21, 24, 25]. Percentage of signal recovery (PSR) and signal recovery (SR) were calculated from mean signal intensity at baseline (S_pre_) and the signal average of 5 consecutive data points obtained 25 s after the mean arrival time of the bolus in the brain (S_post_) as shown in Fig 1. This time point was chosen reflecting the common practice in humans where this measurement is usually performed immediately after the first recirculation of the contrast agent which is about 60 s. With a mean blood volume of 62 ml/kg, rats used in this study had a total blood volume of about 22 ml. With a mean cardiac output of 50 ml/min this results in a corresponding circulation time of about 25 s.

The calculated parameter maps were independently analyzed by two experienced radiologists. For each parameter the time point at which a difference between the two hemispheres was noticed was recorded.

### Histology and immunohistochemistry

Finally, after an overdose of ketamine and medetomidine, rats were transcardially perfused with 4% paraformaldehyde in PBS. Brains were removed, postfixed in 4% paraformaldehyde in PBS overnight, dissected and embedded into paraffin. 2–3 μm sections were cut on a sliding microtome and processed as described previously [26]. Immunohistochemistry using rabbit anti-Von Willebrand factor antibody (Abcam, Cambridge, England) at a dilution of 1:200 was applied to visualize neoangiogenesis. Sections were counterstained with hematoxylin to visualize cell nuclei and to determine the tumor center and tumor rim.

## Results

### Tumor detection and tumor growth over time

**Fig 2:**
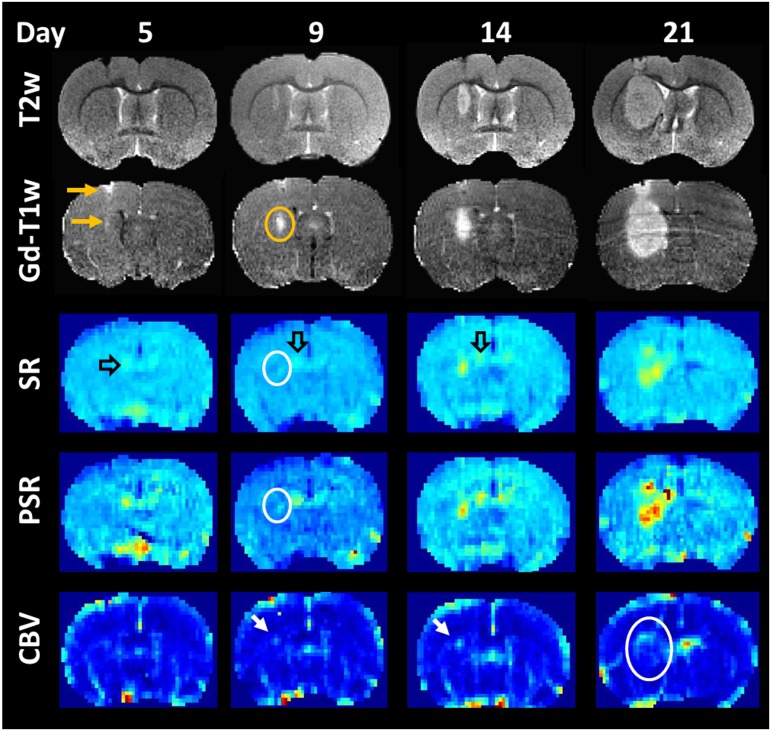
Time course of tumor development after intracerebral injection of 10,000 C6-glioma cells. T2-weighted images (T2w) revealed an increase in tumor mass over time which was accompanied by an increasing area of contrast enhancement (G1-T1w). At day 9 maps of percentage of signal recovery (PSR) and signal recovery (SR) indicated a higher capillary permeability in the tumor center (white ring) extending over time. Higher PSR and SR values were also seen in the region of the choroid plexus (open arrow). Higher cerebral blood volume (CBV) was mainly found at the tumor rim (white arrow and ring).

The time point of first tumor detection was mainly determined by the number of injected C6 cells with the earliest in vivo imaging detection of a tumor on day 5 after tumor cell injection (Table 1). In all cases, tumors were firstly detectable on T2-weighted images as hyperintense regions. Similarly early a signal enhancement on T1-weighted images 10 min after Gd-DTPA was observed (Table 1). The size of the tumor on T2-weighted images increased steadily until the end of the experiment and the leakage of the blood-brain barrier also persisted from first detection until the animal was sacrificed (Fig 2).

### Perfusion and permeability of blood-brain barrier

Changes in tissue blood perfusion and rapid signal enhancement after Gd-DTPA administration were the first time detected 4 to 9 days after the appearance of the tumor on T2-weighted images (Table 1, Fig 2). The tumor volumes at these respective time points ranged between 3 mm^3^ and 53 mm^3^.

Regions in which the signal intensity exceeded the baseline already 25 s after the mean arrival time of the Gd-DTPA bolus were predominantly located in the tumor center and grew continuously with tumor size. In contrast, increase in cerebral blood volume was mainly limited to the tumor rim.

**Fig 3:**
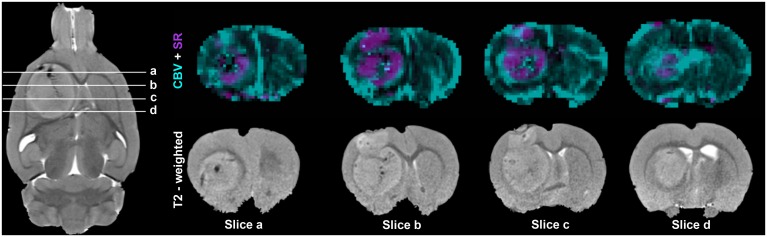
Spatial heterogeneity of cerebral blood volume (CBV) and signal recovery (SR). Maps of SR (purple) were overlaid on maps of CBV (green) obtained on day 21 after injection of 100,000 C6 cells. Areas of increased CBV were mostly found at the tumor rim whereas signal recovery exceeding the baseline was mainly seen in the tumor center excluding regions which were most likely necrotic (dark on T2 weighted images). Coronally and axially oriented T2-weighted images (T2w) are shown as reference.

**Fig 4:**
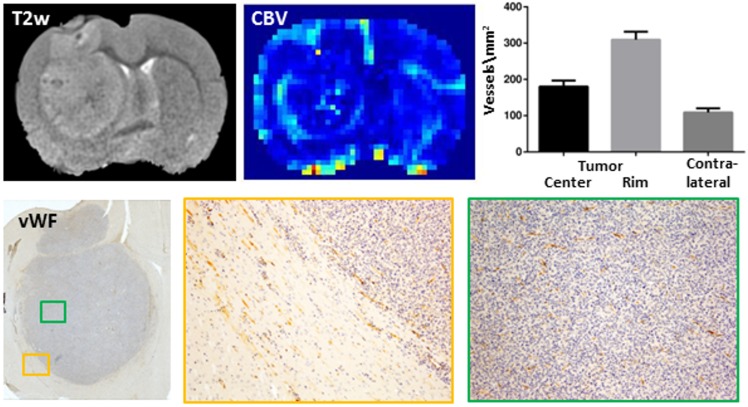
Cerebral blood volume (CBV) and vessel density. The highest CBV was found at the rim of the tumor which also showed the highest vessel density (bar graph) as revealed by immunohistochemistry for von Willebrand factor (vWF, lower row): (left) overview showing the position of the magnified view of the tumor rim (orange box) and tumor center (green box), upper row, left: the corresponding axially oriented T2-weighted image.

Fig 3 shows an overlay of CBV and SR maps obtained 21 days after tumor cell injection. While the permeability of the blood-brain barrier was increased in the central regions, CBV was particularly enhanced at the tumor rim. Histology confirmed a higher blood vessel density at the tumor rim as compared to the tumor center (Fig 4).

### Comparison of PSR and SR maps

**Fig 5:**
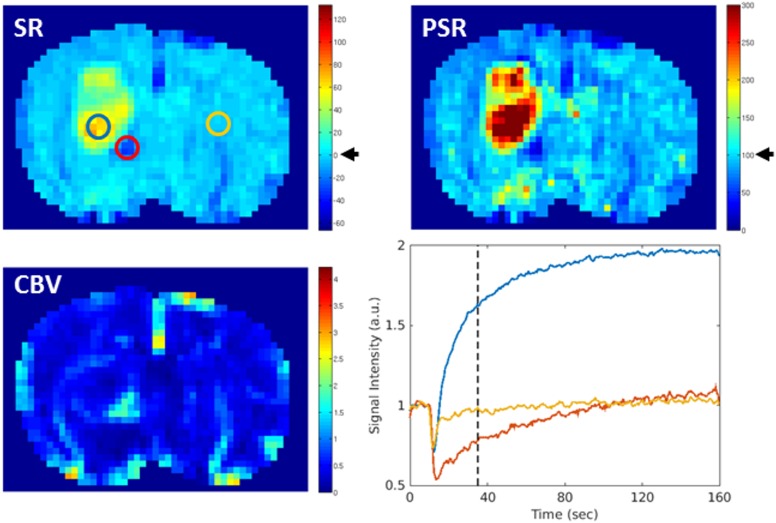
Maps of signal recovery (SR), percentage of signal recovery (PSR) and cerebral blood volume (CBV) in comparison. Recovery of signal intensity at baseline level corresponds to a value of 0% on the SR map and 100% on the PSR map (black arrow). For better comparison the maps were scaled in a way that these two values marked the end of the first third of the entire value range of the respective map. Thus, with the used color coding, regions with a signal increase above baseline appeared yellow to red while those in which the signal intensity did not recover to baseline appeared blue on both maps. The signal-intensity time curve of selected regions of interest (ROI) is shown on the right, lower row. PSR and SR revealed a similar spatial distribution of regions with an increased capillary permeability, with highest level in the tumor center (blue ROI). Low PSR and SR were mostly accompanied by high CBV values (red ROI), whereas the signal intensity on the contralateral side went back to baseline (yellow ROI) within the observation time.

PSR and SR reflect the signal intensity 25 s after the mean arrival time of the Gd-DTPA bolus. This intensity was expressed relative to the signal intensity at baseline. A restoring of signal intensity at baseline corresponds to a value of 100% on the PSR and to 0% on the SR map (Fig 5). PSR and SR showed a similar time course and spatial distribution of increased capillary permeability (Table 1, Figs 2 and 5).

Beside increased PSR and SR values in the center of the tumor, values above the baseline were also observed at the choroid plexus, while larger veins were featured by low PSR and SR values. Moreover, low PSR and SR were mostly accompanied by high CBV values.

**Fig 6:**
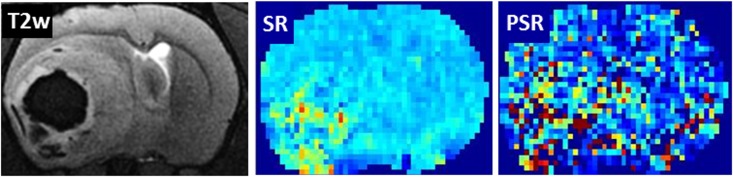
Robustness of signal recovery (SR) and percentage of signal recovery (PSR) maps. In case of alteration of the bolus peak PSR maps became unusable, while SR still provided exploitable results. On the right the corresponding axially oriented T2-weighted image.

In general, PSR maps appeared noisier. In contrast to SR, PSR relies on a precise determination of the signal minimum at contrast agent bolus (Fig 1). At 2 of 23 time points no meaningful PSR map could be calculated due to an alteration of the bolus peak, whereas the SR map provided exploitable results at all time points (Fig 6).

## Discussion

Understanding the time course of tumor development is important for our understanding of tumor progression, for therapy planning and for the development of novel, possibly individualized, cancer treatments. Here, C6 gliomas were initially detectable as regions of signal enhancement on T2-weighted images and similarly early after Gd-DTPA administration on T1-weighted images. Maps of cerebral blood volume (CBV), signal recovery (SR), and percentage of signal recovery (PSR) revealed significant regional differences in blood volume and vascular permeability between tumor center and tumor rim over time.

Onset of glioblastoma development was characterized by a mild leakage of the blood-brain barrier (slow passage of Gd-DTPA within 10 min) without significant neoangiogenesis (unchanged CBV). This type of leakage which was also described in GL261 gliomas [27] might deserve further investigation since it might offer an entrance for targeted drug therapeutics into the brain tumor.

First signs of neoangiogenesis were mainly restricted to the tumor rim, but also with tumor growth higher CBV values were limited to the most peripheral tumor regions. The decrease of the relative blood volume in the former rim may be caused by a considerable increase in tumor cell density, but we cannot fully exclude that this decreasing CBV might be a methodical artifact. Fast extravasation of the contrast agent (within the time scale of bolus arrival) can attenuate the effect of signal reduction by intravascular shortening of T2* and enhance the effect of signal increase by extravascular T1-shortening, both leading to an underestimation of CBV [28]. Given the fact that the vessel density in the tumor center was lower than at the tumor rim, but higher than in the control region, both effects may play a role here. Several human studies showed a correlation between CBV values of the tumor and histologic grading of gliomas [5, 6, 29] with increasing CBV in higher grades. In human glioblastoma high CBV values also dominate the tumor rim, but were in some cases surrounded by hypoperfused areas [30]. Glioblastoma is a highly vascularized neoplasm in which angiogenesis is supposed to be triggered by the expression of the hypoxia-inducible factor (HIF-1) and by vascular endothelial growth factor (VEGF) secreted by the tumor cells [31] and the effects are further enhanced by a variety of pro-angiogenic cytokines. In part, these processes are, however, countered by multiple anti-angiogenic cytokines of the tumor-microenvironment inhibiting neovascularization [32]. Finally, regions of an increased CBV may therefore indicate successful invasion of the tumor cells. However, to answer the question whether and how the spatial changes in CBV may predict tumor cell invasion and the direction of tumor dissemination further studies are required.

Four to 9 days after tumor detection the capillary permeability significantly increased in the tumor center as observed by a fast signal enhancement already 25 s after Gd-DTPA bolus (PSR >100%, SR >0%). This increase in capillary permeability most likely reflects the beginning of necrosis within the tumor center, which is a hallmark of glioblastoma [33]. The leakage of blood brain barrier in the neo-angiogenic rim, however, was significantly less pronounced. This suggests that the newly formed microvasculature in the rim may lack mature tight junctions but still ensured sufficient oxygen and nutrients supply for the growing tumor.

Moreover, the strong leakage of the blood brain barrier in the tumor center but only mild opening in the tumor rim may be of particular importance for any type of drug therapy because it might indicate lower extravascular drug availability in the relevant region of tumor progression at the rim.

PSR larger 100% is not frequently seen in human glioblastoma [10–12, 34]. In fact, it has been proposed that high PSR may indicate primary CNS lymphoma whereas low PSR may indicate glioma [10–12, 34]. This may partially be explained by differences between the animal model and the human disease. In addition, differences in MR-parameters and magnetic field strengths may hamper a direct comparison. However, it is a common practice in human diagnostics to focus on regions of interest located particularly in areas of high CBV [12, 35]. In view of the significant heterogeneity described here, this approach may bear the risk of overlooking regional differences. In fact, also in this study regions with high CBV did not show fast signal enhancement (increased brain capillary permeability).

When comparing results of animal study with observations in human patients, also other confounders may in addition play a role. Particularly therapeutic interventions including surgery, radiation therapy, and chemotherapy [3] can influence brain perfusion and capillary permeability. Its sensitivity to therapy makes MRI perfusion measurement, however, a very valuable diagnostic tool. It has been shown that DSC-MRI can distinguish between progression and pseudo progression [36] and therefore adds valuable information to the RANO-criteria [37] being the standard criteria currently used to measure the response to glioma treatment.

PSR and SR strongly depend on the applied MR-parameters and in addition, particularly SR may be affected by strong changes in CBV, and DCE-MRI may be superior to DSC-MRI when assessing capillary permeability [7]. However, PSR and SR maps can be rapidly acquired and are technically very easy to realize. SR even does not require determination of the bolus peak, which makes it a robust and easy to obtain parameter. PSR and SR maps provided very similar results but SR maps were less sensitive to motion or sharpness of the contrast agent bolus. Moreover, SR maps provide information about the spatial distribution of early signal enhancement and may replace the ROI analysis of the signal intensity time curve commonly used clinical diagnostic.

## Conclusions

Tumor progression in C6 glioblastoma was characterized by temporal and spatial changes in CBV and capillary permeability. Particularly CBV and the signal enhancement immediately after the first recirculation of the contrast agent showed substantial regional differences within the tumor mass emphasizing the value of parameter maps in contrast (or in addition to) region-of-interest analyses. Since DSC-perfusion is a fast imaging procedure, which already is part of MRI-protocols for glioblastoma, the proposed SR maps may ad valuable diagnostic information without requiring additional measurements. However, further studies may be needed to evaluate the value of SR maps in human patients.
